# Goats and Homœopathy

**Published:** 1883-01

**Authors:** 


Goats and Homœopathy.—It is a pleasure to learn that the goat is an animal which responds well to homœopathic remedies. In Surrey, England, there is a goat farm where Guilelmus capricornis is raised and milked for the alleged benefit of the babies of London. A visitor says: "The goats, young and old, appear clean and perfectly healthy; their bright, hairy coats are subjected to curry-combing; no troublesome foot disease demands attention as in the case of sheep, and any internal ailments are promptly and successfully dealt with by homœopathic medicines, of which the manager, Mr. Farrer, speaks with the greatest confidence and satisfaction."

We should like very much to have Mr. F. report to us some cases. No doubt infinitesimal doses will affect a credulous man, but not a sensible goat.

